# Broadband generation of accelerating polygon beams with large curvature ratio and small focused spot using all-dielectric metasurfaces

**DOI:** 10.1515/nanoph-2021-0787

**Published:** 2022-02-15

**Authors:** Lei Chen, Saima Kanwal, Yongzheng Lu, Dawei Zhang, Xu Chen, Jian Chen, Jing Wen

**Affiliations:** Engineering Research Center of Optical Instrument and Systems, Ministry of Education and Shanghai Key Lab of Modern Optical System, University of Shanghai for Science and Technology, No. 516 Jun Gong Road, Shanghai, 200093, China; School of Optical-Electrical and Computer Engineering, University of Shanghai for Science and Technology, Shanghai, 200093, China

**Keywords:** accelerating beam, large curvature ratio, metasurface, polygon beam, small focused spot

## Abstract

Self-accelerating polygon beams have drawn growing emphasis in optics owing to their exceptional characteristics of multiple self-accelerating channels and needle-like field distributions. Various approaches have been proposed to generate polygon beams, such as using spatial light modulators (SLMs) or plasmonic metasurfaces. However, SLMs impede the miniaturization of the optical system and both approaches are subject to low efficiencies and demand an extra physical lens with a long focal length for Fourier transform, which limits the quality and the diverse variability of polygon beams. In this article, we demonstrate the generation of high-quality accelerating polygon beams in broadband spectra of 500–850 nm by utilizing dielectric metasurfaces. These metasurfaces integrate the functionality of the Fourier transform lens to enable the resulting beams with a large curvature ratio for the self-accelerating channels and a relatively small size for the autofocus region. The curvature ratio of the beam at *λ* = 633 nm is 31 times higher than the previously reported plasmonic-based method. While the size of the focused spot is 2.35 µm, which is reduced by nearly 15 times. The proposed beam generator provides ample opportunities for applications such as particle micromanipulation, beam shaping, laser fabrication, and biomedical imaging.

## 1 Introduction

At the outset of the experimental measurement of the Airy optical beam [1], the accelerated beams that travel in curved lines in free space have gained enormous research interest. Previous studies have focused on various types of accelerating beams such as accelerating light beams along arbitrary convex trajectories [2], nonparaxial Mathieu and Weber beams [3], spherical shaping of light [4], Bessel-like optical beams with arbitrary trajectories [5], and abruptly autofocusing beams [6]. Compared to the above accelerating beams, polygon beams (PBs) possess unique characteristics of not only multiple self-accelerating channels with cusp points arranged in a circle but also needle-like auto-focused field distributions with fewer side lobes relative to Bessel beams [7], [8], [9]. Following the current theoretical demonstration of the finite-energy accelerating PBs [7], experimental demonstration of various types of PBs has been reported as well [8, 9].

Contemporary methods utilized to generate polygonal beams require an additional physical lens to perform Fourier transform [7–10], which poses a challenge to its integration in a compact nanophotonics system. Above all, it is essential to reduce the focal length of the Fourier transform lens to generate high-quality PBs with various beam parameters, e.g., PBs with a high curvature ratio for multiple self-accelerating channels or a needle field with a relatively small size for the autofocus region. Though the minimum focal length of the conventional commercial objective lens limits feasible control parameters for beam generations, thus the diverse variability of PBs. Therefore, it is necessary to combine the K-space phase and the lens phase for Fourier transform to generate the PBs. Meanwhile, there is a pressing need for devices with subwavelength pixel sizes for controlling light fields. For instance, designing lenses with shorter focal lengths is limited by the Nyquist sampling theorem (the Nyquist focal length can be described as *f*_N_ = *N*Δ^2^/*λ*, where *N* is the number of pixels in a row and Δ is the pixel size [11]).

Over the past few years metasurfaces, which are artificially engineered of subwavelength planar nanostructure arrays, have opened up a new era [12, 13] for engineering optics due to the rapid development of optical functional meta-devices including near-diffraction-limited or achromatic metalenses [14], [15], [16], [17], [18], quantum photonics [19], [20], [21], [22], metaholograms [23, 24], high harmonic generation in nonlinear optics [25, 26], manipulating self-accelerating beams [27], [28], [29], [30], [31], [32], [33], [34], [35], [36], and so on [37, 38]. Furthermore, all-dielectric metasurfaces exhibiting miniaturization and integration of the optical system have made substantial advancements in self-accelerating beams which include Airy beams and Bessel-like beams but not PBs yet [28], [29], [30], [31], [32], [33], [34], [35], [36]. Although plasmonic metasurfaces have been employed to generate optical PBs [39], the strong ohmic losses of the metals at the visible regime significantly limit the efficiency and quality of the beams. Hence, we employed a combination of the K-space phase used to effectively generate the PBs and the phase of the Fresnel holographic lens and encoded it on the all-dielectric metasurface to generate high-quality PBs, featuring multiple self-accelerating channels with a large curvature ratio and a needle-like field with a small transverse size. Attributed to the higher refractive index and minimal absorption losses of the silicon, our designed device is remarkably efficient in the broadband regime of visible to near-infrared light, i.e., 500 nm to 850 nm. The measured generation efficiency of the device is as high as 43.1% at *λ* = 722 nm, which is approximately 15 times higher. And the curvature ratio of the beam is as large as 31 times higher than that of the recently reported plasmonic metasurfaces [39]. Simultaneously, the needle-like autofocused beam retains the full width at half-maximum (FWHM) as small as 2.35 μm (3.71*λ*, *λ* = 633 nm) at the focal plane, which is reduced by nearly 15 times [39]. We envision this work would inspire the creation of ultra-compact planar nanophotonics platforms for efficient generation of PBs and has great potential for the application scenarios such as particle manipulation [10], laser fabrication [40], and biomedical imaging [41].

## 2 Methodology

The electric field of PBs in an imaging system of focal length of *f* with an incident plane wave can be described in the Cartesian coordinates [7, 8, 39]:

(1) $$\begin{array}{l} E\left(x,y,z\right)=A\overset{\infty }{\underset{-\infty }{\iint }}\text{exp}\left[ik\Psi \left(x\prime ,y\prime ,x,y,z\right)\right]dx\prime dy\prime \end{array}$$

where *A* is the constant, *Ψ*(*x*′, *y*′, *x*, *y*, *z*) = *ϕ*_*m*_ (*x*′, *y*′) − *z*(*x*′^2^ + *y*′^2^) − (*x*′*x* + *y*′*y*) is determined by the phase function, *k* = 2π/*λ* is the wave number, (*x*′, *y*′) denote the transverse coordinates in the input plane and (*x*, *y*, *z*) represent the coordinates in the propagation plane. The phase *ϕ*_*m*_(*x*′, *y*′) that generates PBs in the K space can be written as:

(2) $$\begin{array}{l} \phi_{m}\left(x\prime ,y\prime \right)=C{\left(x\prime^{2}+y\prime^{2}\right)}^{\frac{m}{2}}\;\sin\left(m\times a\text{tan}\left(\frac{y\prime }{x\prime }\right)+\omega \right) \end{array}$$

where *C* and *ω* represent a constant scaling number and the initial phase, respectively, and the polynomial order *m* represents the order of PBs.

**Figure 1:**
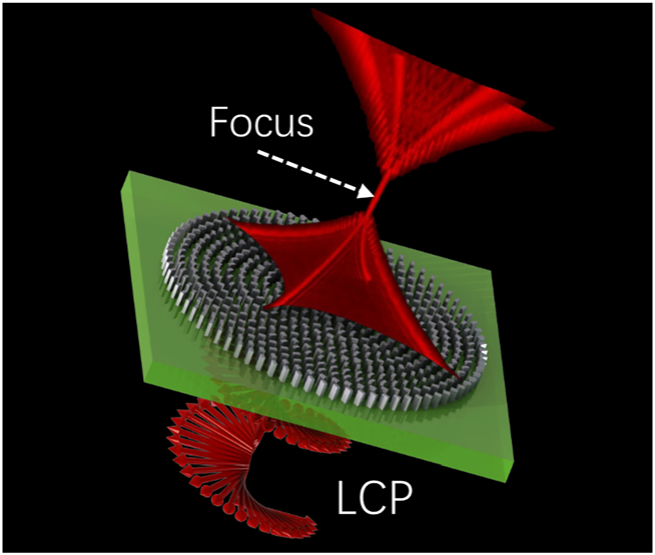
Schematic of all-dielectric metasurface for generating PBs. The LCP light is incident to the metasurface and the transmitted inversed polarized light will be shaped to multiple accelerating channels and needle-like auto-focused field distribution.

Here, the phase of the Fresnel holographic lens and the phase used to generate PBs in the K space is simultaneously loaded on the metasurface to generate PBs, as shown in Figure 1. When the left circularly polarized (LCP) light is converted into inverse circularly polarized light, the transmitted inversed polarized light will be shaped to multiple accelerating channels and a needle-like auto-focused beam. The Fresnel holographic lens replaces the traditional physical lens for Fourier transform which can significantly reduce the system’s size. This method is also known as the Fresnel holographic lens method and has been experimentally demonstrated in Airy beams [34]. The synthetic phase imparted by the metasurface to generate PBs is given as:

(3) $$\begin{array}{l} \varphi_{m}\left(x\prime ,y\prime \right)=C{\left(x\prime^{2}+y\prime^{2}\right)}^{\frac{m}{2}}\;\sin\left(m\times \text{atan}\left(\frac{y\prime }{x\prime }\right)+\omega \right)-\frac{\pi }{\lambda f}\left(x\prime^{2}+x\prime^{2}\right) \end{array}$$

where *f* is the focal length of the Fresnel holographic lens. To validate our demonstrated method, subsequent experiments were carried out for the PBs with 4 and 6 self-accelerating channels at the design wavelength i.e., *λ*_d_ = 650 nm.

**Figure 2:**
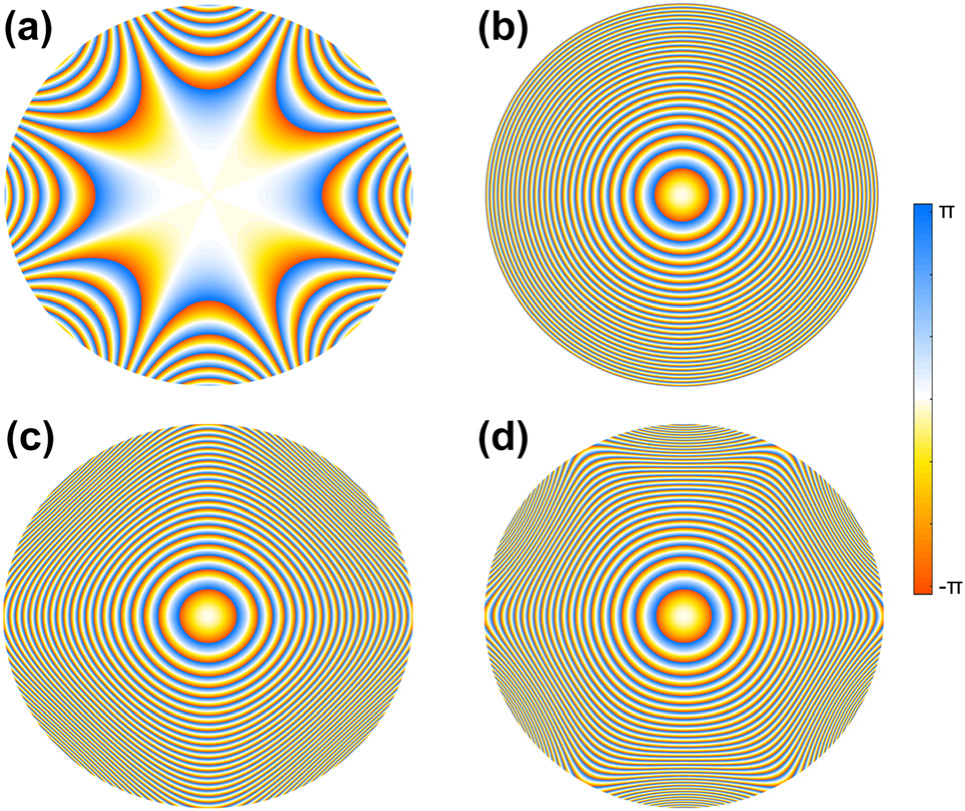
Phase design for generating PBs. (a) The phase profile used to generate the PB with *m* = 4 in the K space. (b) The phase profile of the Fresnel lens. (c) and (d) The phase profiles to generate the PBs with *m* = 4 and *m* = 6 in the real space, respectively.

Figure 2(a) shows the typical phase profile used to generate the PB in the K space with *m* = 4, *C* = 1/(3 × 10^6^), and *w* = *π*/2. The diameter of the phase map is 200 μm. The phase of a Fresnel holographic lens with a focal length *f* = 300 μm utilized for Fourier transform is shown in Figure 2(b). While Figure 2(c) presents the synthetic-phase mask (addition of Figure 2(a) and Figure 2(b)) imposed on the designed metasurface to generate the PB with four self-accelerating channels. Likewise, Figure 2(d) is also the synthetic-phase mask to generate the PB with *m* = 6, *C* = 1/(3 × 10^10^), *w* = *π*/2 and *f* = 300 μm.

## 3 Design of all-dielectric metasurface

**Figure 3:**
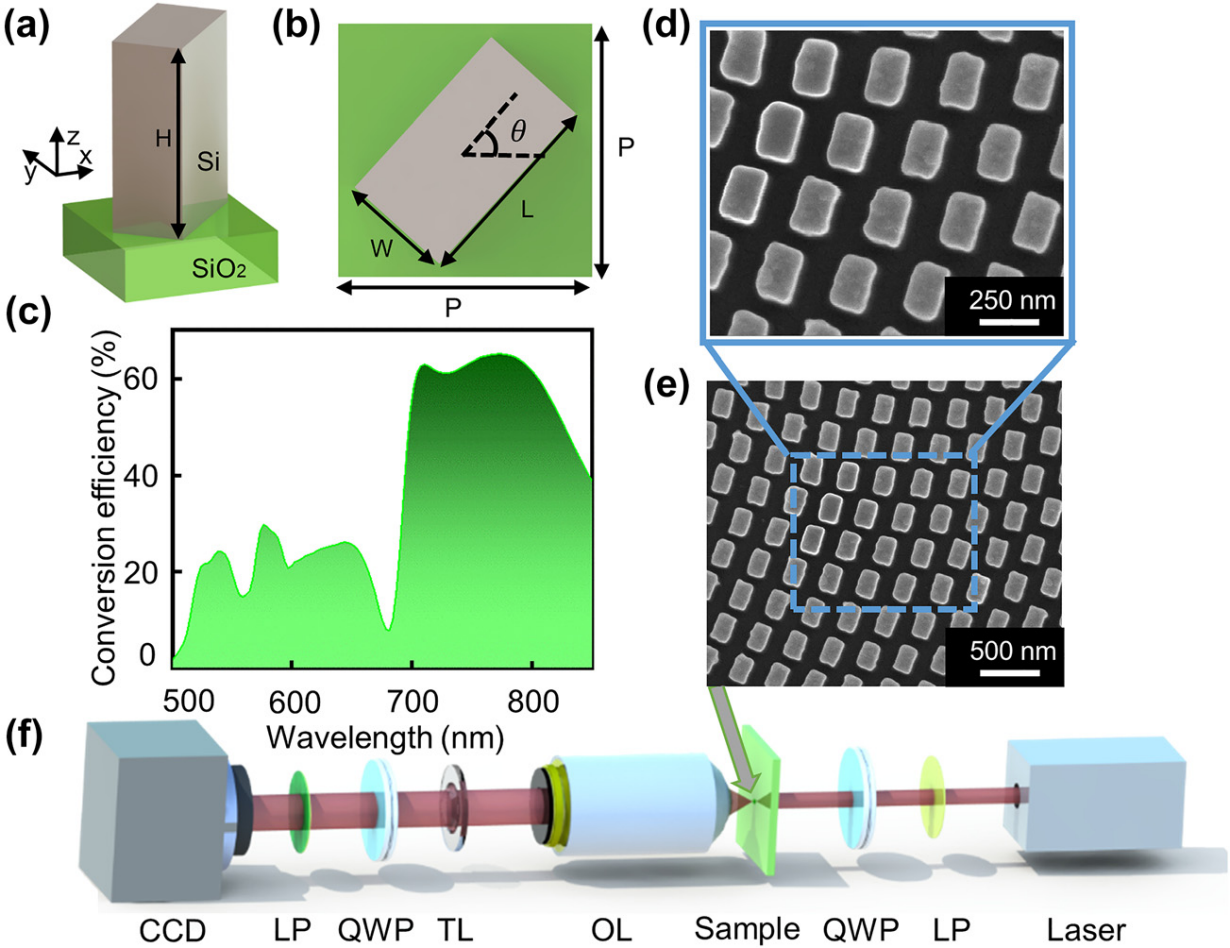
Nanopillar design and experimental setup. (a) The building block of the metasurface consists of amorphous Si nano-fin sitting on a silica substrate. (b) Top view of the building block with nano-fin length *L* = 240 nm, width *W* = 135 nm, and height *H* = 365 nm. The nano-fin can rotate in the *x*–*y* plane with an angle *θ* to realize the required phase according to the Pancharatnam–Berry phase with a pitch *P* = 300 nm. (c) The simulated polarization conversion efficiency (defined as the ratio between the total optical power of the cross-polarized light to the input optical power) of the unit cell. The expanded (d) and top-view (e) scanning electron microscopy (SEM) images of a portion of the fabricated sample. (f) Experimental setup for the optical characterization (quarter-wave plate (QWP), linear polarizer (LP), objective lens (OL), tube lens (TL), and charge-coupled device (CCD)).

The unit cell of the designed metasurface is composed of amorphous silicon (Si) nano-fins with height *H*, length *L,* and width *W* deposited on a silica substrate with square lattice as shown in Figure 3(a) and (b). All the simulations were carried out using the commercial finite-difference-time-domain (FDTD) software developed by Lumerical Inc. [42]. To acquire the maximum polarization conversion efficiency periodic boundary conditions were applied to *x* and *y*-direction while perfectly matched layer boundary conditions were applied to the *z*-direction for the broadband regime of *λ* = 500–850 nm. The optimized structure parameters for the unit cell are: height *H* = 365 nm, width *W* = 135 nm, length *L* = 240 nm and the lattice constant *P* × *P* = 300 nm × 300 nm. The simulated polarization conversion efficiency of the fabricated sample is as high as 26% at the design wavelength *λ*_d_ = 650 nm and above 50% in the wavelength regime of *λ* = 697–826 nm, as shown in Figure 3(c). Moreover, it is experimentally validated in the next section that the high-quality accelerating PBs are generated in the entire broadband regime of *λ* = 500–850 nm. To accomplish the required phase profiles the Pancharatnam–Berry phase [43] method was employed in the unit cell. Figure 3(d) and (e) displays the scanning electron microscopy (SEM) images of a portion of the fabricated sample in top view. Figure 3(f) shows the experimental setup of the light field characterization (see the measurement details in Experimental section).

## 4 Results

**Figure 4:**
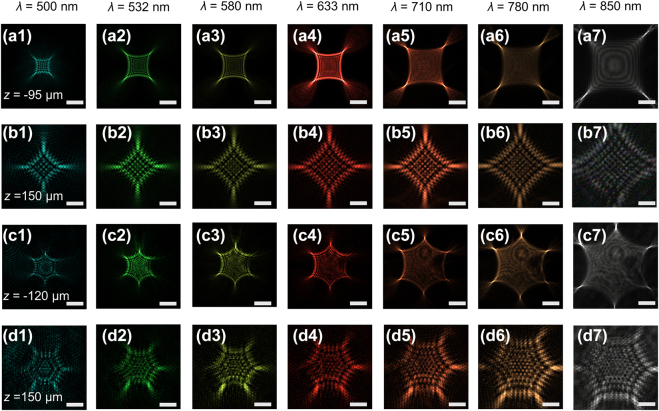
Measured intensity distributions of the PBs in *x*–*y* plane. Measured intensity distributions of the generated quadrilateral beams (a1)–(a7) for *z* = −95 μm and (b1)–(b7) for *z* = 150 μm at wavelengths of *λ* = 500, 532, 580, 633, 710, 780 and 850 nm, respectively while, measured intensity distributions of the generated hexagon beams (c1)–(c7) for *z* = −120 μm and (d1)–(d7) for *z* = 150 μm at wavelengths of *λ* = 500, 532, 580, 633, 710, 780, and 850 nm, respectively. Scale bar = 17 μm (a1)–(a7), (b1)–(b7) and 20 μm (c1)–(c7), (d1)–(d7).

We have experimentally demonstrated PBs with relatively large curvature ratios for multiple accelerating channels and narrow beam widths for needle-like auto-focused field distributions. As a proof of concept demonstration, we show the generation of the PBs based on metasurfaces with *m* = 4 and *m* = 6. The phase distribution imposed on the metasurface corresponding to the optical field distribution of the quadrilateral beams with *m* = 4 as shown in Figure 4(a1)–(a7) and (b1)–(b7) and the hexagonal beams with *m* = 6 as shown in Figure 4(c1)–(c7) and (d1)–(d7) are shown in Figure 2(c) and (d), respectively. The measured intensity distributions of quadrilateral beams with *m* = 4 and hexagonal beams with *m* = 6 at *λ* = 500, 532, 580, 633, 710, 780 and 850 nm at various propagation distances are shown in Figure 4 (*z* = −95 μm for Figure 4(a1)–(a7), *z* = −120 μm for Figure 4(c1)–(c7) and *z* = 150 μm for Figure 4(b1)–(b7) and (d1)–(d7). The corresponding theoretical results are shown in Figure S1. The intensity pattern of each PB is homogenous for different wavelengths, with an expansion in size as the wavelength increase. This phenomenon is caused by the chromatic aberration of the Fresnel metalens. The focal length of the lens decreases with an increase in the wavelength so the intensity distributions of PBs keep stirring away from the optical axis since the wavelength increases when the phase used to generate the polygonal beam remains unchanged.

As illustrated in Figure 1, the three-dimensional intensity patterns are evolved into focus of a singular point from a polygon with intensive cusps and then splits into four or six off-axis identical lobes. The singular point appears on the focal plane of the Fresnel lens (*z* = 0 µm) and then the PBs are generated during propagation after the focal plane. The self-accelerating trajectories in front and back of the focal plane are identical in number while differing in the direction of transverse deflection of the beam when comparing Figure 4(a1)–(a7) at *z* = −95 μm, Figure 4(c1)–(c7) at *z* = −120 μm and Figure 4(b1)–(b7) and (d1)–(d7) at *z* = 150 μm.

**Figure 5: j_nanoph-2021-0787_fig_005:**
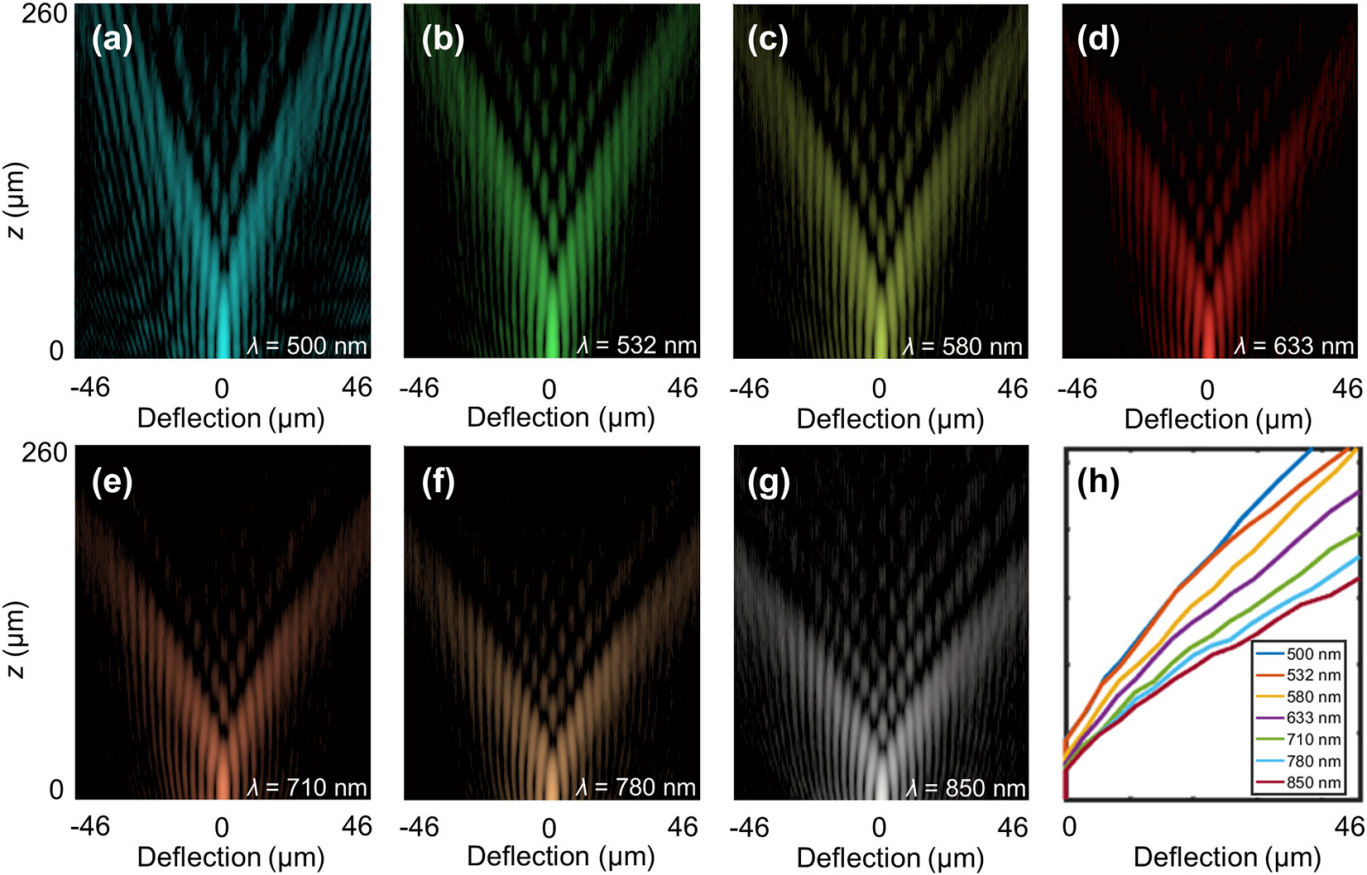
Measured longitudinal intensity distributions of the PB. (a)–(g) Measured longitudinal intensity distributions for the PB with *m* = 4 at wavelengths of *λ* = 500, 532, 580, 633, 710, 780, and 850 nm, respectively. To facilitate visualization, the intensity distribution in (a)–(g) are false-colored images in the log scale. (h) Measured beam deflection of the main accelerating channel along the longitudinal direction for the above wavelengths.

The focal length of the Fresnel lens, used for the Fourier transform in our design approach of the synthetic-phase metasurface, can be theoretically set to a relatively small value without considering the alignment issue of the optical axis. The Nyquist focal length is *f*_N_ = *N*Δ^2^/*λ* = 95 μm, where *N* = 666, Δ = 300 nm (*λ* = 633 nm) [11]. The resulting PB with *m* = 4 has a large curvature ratio for multiple accelerating channels and a small needle-like auto-focused field. To demonstrate the above properties, first, the *xz* cross-section of the intensity distribution (see optical characterization in Experimental section) is reconstructed for the PB with *m* = 4 at wavelengths of *λ* = 500, 532, 580, 633, 710, 780, and 850 nm, respectively, as shown in Figure 5(a)–(g). Then the beam deflection of the main accelerating channel along the longitudinal direction is shown in Figure 5(h). The corresponding theoretical results are shown in Figure S2. The curvature ratio is defined as the ratio of the deflection distance along the transverse direction to the propagation distance along the longitudinal direction. It is noted that the curvature ratio of the PB with *m* = 4 is 0.193 (29 μm from the optical axis at *z* = 150 μm), which is as large as 31 times higher than that of the recently reported plasmonic metasurface (about 0.375 mm from the optical axis at *z* = 60 mm) at the wavelength of 633 nm [39]. As the wavelength increases, the curvature ratio also increases as shown in Figure 5(h).

**Figure 6:**
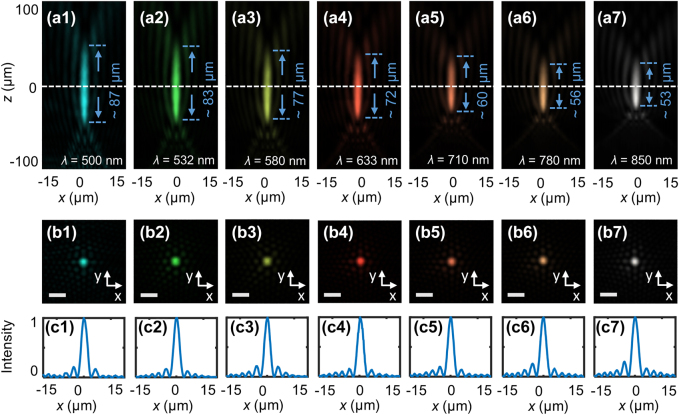
Measured intensity distributions of the PB in the needle-like region. (a1)–(a7) Intensity distributions of the PBs (*m* = 4) along the optical axis (*x*–*z* plane) and (b1)–(b7) measured transverse field distributions (*x*–*y* plane) at the focal plane of the PBs at wavelengths *λ* = 500, 532, 580, 633, 710, 780, and 850 nm. To facilitate visualization, the intensity distributions in (a1)–(a7) are false-colored images, because these intensity distributions are extracted from the reconstructed three-dimensional image, while the intensity distributions in (b1)–(b7) are true-colored images. (c1)–(c7) Normalized intensity profiles along the horizontal axes at the center of the focal spots. Scale bar = 6 μm.

Regarding the traditional methods for PBs generation, as the working wavelength changes, the position of the physical objective lens must be adaptively adjusted since the physical lens usually has a large dispersion. Our designed synthetic-phase metasurfaces evade this issue efficiently due to the large operating bandwidth of the device. To further validate the wideband self-focusing characteristics of the PBs with relatively small transverse sizes, Figure 6(a1)–(a7) and (b1)–(b7) manifest the reconstructed longitudinal needle-like field distributions and the extracted transverse fields at the focal plane of the metasurface (the white dashed line in Figure 6(a1)–(a7)) at the wavelengths of *λ* = 500, 532, 580, 633, 710, 780, and 850 nm, respectively. Figure 6(c1)–(c7) depict the normalized intensities along the horizontal axis at the center of the focal plane corresponding to Figure 6(b1)–(b7), and the FWHMs at the wavelengths of 500, 532, 580, 633, 710, 780, and 850 nm are 2.36, 2.25, 2.25, 2.35, 2.21, 2.27, and 2.39 μm, respectively. The corresponding theoretical results are shown in Figure S3. The experimental results realize an ultra-high quality of the spot due to the high refractive index and lower absorption losses of the silicon at the target wavelength *λ* = 500 nm (3.92 + 0.12i) to 850 nm (3.34 + 0i). It is worth noting that at *λ* = 633 nm, the beam has a focused spot size of 2.35 μm (3.71*λ*) which is reduced by nearly 15 times [39], and a focal depth of about 72 μm (114*λ*). Meanwhile, the side lobes of the beam are much smaller than the Bessel beam, as Bessel beams have high-energy side lobes [44]. Figures S4 and S5 shows that the length of the needle-like region increases as *C* increases when the focal length *f* of the Fresnel lens is determined, which is unlike the traditional method to control needle region through optimization algorithm [45, 46]. Figure S6 shows the simulated polarization of electric fields and energy flow states in the needle region of the PB. Airy beam and the tailored Bessel beam have achieved great success in the field of biological microscopy imaging [41] and laser processing [47, 48], and we envision that the generated PBs with long depths of focus in the needle regions bear great potential in light-sheet microscopy, high-aspect-ratio through-silicon vias, and such other applications. In addition, we simulate the PB with *m* = 1. As shown in Figure S7(d), the PB spreads to both sides along the inclined straight line when propagating in the *x*–*z* plane. We expect this work to stimulate research interest in further types of PBs.

The measured generation efficiency of the PB (defined as the ratio of the intensity of the generated PBs to that of the incident beam) is as high as 39.83% (*m* = 4) at *λ* = 722 nm in the plane of *z* = 150 μm. Here, the measured generation efficiency is *p* = *I*_1_/*I*_0_, where *I*_1_ and *I*_0_ are obtained by integrating the grey values of each pixel in the light field distribution of the PB and that of the laser through the substrate only, captured by the CCD. Using the above method, the measured efficiencies are 38.22%, 41.21%, 42.49%, and 43.10% at *z* = 175 μm, 125 μm, 105 μm, and 90 μm, respectively. This is extremely critical for generating high-quality PBs and further enhancing the feasibility of such beams. The experimentally measured high-quality PBs validate that the device is capable of achieving high-efficiency and broadband generation of PBs from visible to the near-infrared band while conventional methods, based on SLMs or plasmonic metasurfaces [7–10], are challenging to implement.

## 5 Conclusions

In summary, to overcome the limitations of traditional SLMs or plasmonic metasurface, we demonstrate a highly efficient approach to generate PBs with different self-accelerating channels in a single device for miniaturization and integration of the optical system and devices. Besides, the generated beam has a large curvature ratio for the accelerating channel and a small-sized needle-like focused field. The efficiency of the device is as high as 43.1% at the wavelength *λ* = 722 nm. Substituting the bulky system of Fourier transform for the generation of PBs, the proposed device facilitate a low-cost and ultrathin platform for PBs generation which will be readily integrated with on-chip devices for future applications like high-resolution biological microscopy imaging, optical tweezing of live samples, and laser processing.

## 6 Experimental section

*Sample fabrication*: Electron beam (e-beam) lithography and inductively coupled plasma dry etching process was employed to realize the metasurfaces. Our design utilized silicon as the optical material since it ensures relatively higher polarization conversion efficiency with a rigorous optimization of geometric parameters of the nano-fin. To start with, a 365 nm thick uniform amorphous silicon layer was deposited by a sputtering machine. Then a layer of e-beam resist (CSAR) with a thickness of 200 nm was spin-coated on the wafer afterward. The wafer was baked at 180° for 1 min. E-beam lithography was implemented based on the electron beam writing system (JEOL JBX-9500) with an acceleration voltage of 100 keV. The sample was then developed in the developer AR-600-546 at room temperature for 90 s. Finally, STS advance silicon etching machine was used for silicon dry etching.

*Optical characterization*: The experimental setup for the measurement of field distributions of the generated PBs is illustrated in Figure 3(e). The intensity distributions of the PBs shown in Figures 4 [Fig j_nanoph-2021-0787_fig_005]–6, were imaged using an optical setup that includes an Olympus objective (MPlanFL N NA = 0.45, 20 ×) (to ensure that the resulting image can be entirely detected by the CCD), a tube lens with a focal distance of 180 mm and a CCD camera (DH-HV3151UC) with a resolution of 2048 × 1536 pixels. The metasurface was illuminated by LCP light generated by a linear polarizer (Thorlabs LPVIS100) and a quarter-wave plate (Thorlabs AQWP05M-600) with the incident light generated from the NKT super-continuum light source (NKT Photonics SuperK EXTREME EXR-15). Another quarter-wave plate and polarizer were used to eliminate background light in the optical path i.e., the LCP light. The objective lens and the tube lens were fixed on a high-speed motorized *xy* scanning stage (Thorlabs MLS230-1). The *xz* cross-section of the intensity distribution was obtained by moving the objective lens and the tube lens along the *z*-direction by the *xy* scanning stage at a step size of 1 μm and then reconstructing to 3D field patterns in the longitudinal plane, from which the longitudinal *xz* field distributions of the 2D PBs were extracted as shown in Figure 6(a1)–(a7). The broadband response of the device can be observed by sweeping the wavelength of the super-continuum laser.

## Supplementary Material

Supplementary Material

## References

[1] Siviloglou G., Broky J., Dogariu A., Christodoulides D. (2007). Observation of accelerating Airy beams. *Phys. Rev. Lett.*.

[2] Froehly L., Courvoisier F., Mathis A. (2011). Arbitrary accelerating micron-scale caustic beams in two and three dimensions. *Opt. Express*.

[3] Zhang P., Hu Y., Li T. (2012). Nonparaxial Mathieu and Weber accelerating beams. *Phys. Rev. Lett.*.

[4] Mathis A., Courvoisier F., Giust R. (2013). Arbitrary nonparaxial accelerating periodic beams and spherical shaping of light. *Opt. Lett.*.

[5] Chremmos I. D., Chen Z., Christodoulides D. N., Efremidis N. K. (2012). Bessel-like optical beams with arbitrary trajectories. *Opt. Lett.*.

[6] Zhang P., Prakash J., Zhang Z. (2011). Trapping and guiding microparticles with morphing autofocusing Airy beams. *Opt. Lett.*.

[7] Barwick S. (2010). Accelerating regular polygon beams. *Opt. Lett.*.

[8] Yun-Tian Z., Zhi-Gang Z., Teng C., Qing-Chuan Z., Xiao-Ping W. (2015). Accelerating generalized polygon beams and their propagation. *Chin. Phys. Lett.*.

[9] Gong L., Liu W.-W., Ren Y.-X., Lu Y., Li Y.-M. (2015). Self-bending symmetric cusp beams. *Appl. Phys. Lett.*.

[10] Liu W., Yang X., Gao J. (2019). Optical transportation and accumulation of microparticles by self-accelerating cusp beams. *Phys. Rev.*.

[11] Gutierrez B. K., Davis J. A., Moreno I., Cottrell D. M. (2019). Encoding lenses with focal lengths lower than the Nyquist limit using high phase-modulation displays. *Opt. Lett.*.

[12] Luo X. (2018). Engineering optics 2.0: a revolution in optical materials, devices, and systems. *ACS Photonics*.

[13] Luo X. (2019). Subwavelength artificial structures: opening a new era for engineering optics. *Adv. Mater.*.

[14] Wang S., Wu P. C., Su V.-C. (2018). A broadband achromatic metalens in the visible. *Nat. Nanotechnol.*.

[15] Kanwal S., Wen J., Yu B. (2020). High-efficiency, broadband, near diffraction-limited, dielectric metalens in ultraviolet spectrum. *Nanomaterials*.

[16] Yu B., Wen J., Chen X., Zhang D. (2019). An achromatic metalens in the near-infrared region with an array based on a single nano-rod unit. *APEX*.

[17] Kanwal S., Wen J., Yu B. (2020). Polarization insensitive, broadband, near diffraction-limited metalens in ultraviolet region. *Nanomaterials*.

[18] Khorasaninejad M., Chen W. T., Devlin R. C., Oh J., Zhu A. Y., Capasso F. (2016). Metalenses at visible wavelengths: diffraction-limited focusing and subwavelength resolution imaging. *Science*.

[19] Stav T., Faerman A., Maguid E. (2018). Quantum entanglement of the spin and orbital angular momentum of photons using metamaterials. *Science*.

[20] Wang K., Titchener J. G., Kruk S. S. (2018). Quantum metasurface for multiphoton interference and state reconstruction. *Science*.

[21] Li L., Liu Z., Ren X. (2020). Metalens-array–based high-dimensional and multiphoton quantum source. *Science*.

[22] Solntsev A. S., Agarwal G. S., Kivshar Y. S. (2021). Metasurfaces for quantum photonics. *Nat. Photonics*.

[23] Ren H., Fang X., Jang J., Bürger J., Rho J., Maier S. A. (2020). Complex-amplitude metasurface-based orbital angular momentum holography in momentum space. *Nat. Nanotechnol.*.

[24] Ding X., Wang Z., Hu G. (2020). Metasurface holographic image projection based on mathematical properties of Fourier transform. *PhotoniX*.

[25] Li G., Zhang S., Zentgraf T. (2017). Nonlinear photonic metasurfaces. *Nat. Rev. Mater.*.

[26] Ma M., Li Z., Liu W. (2019). Optical information multiplexing with nonlinear coding metasurfaces. *Laser Photon. Rev.*.

[27] Chen L., Ren T., Zhao Y. (2020). Polarization‐independent wavefront manipulation of surface plasmons with plasmonic metasurfaces. *Adv. Opt. Mater.*.

[28] Ju Z., Wen J., Shi L. (2021). Ultra‐broadband high‐efficiency Airy optical beams generated with all‐silicon metasurfaces. *Adv. Opt. Mater.*.

[29] Guo Y., Huang Y., Li X. (2019). Polarization‐controlled broadband accelerating beams generation by single catenary‐shaped metasurface. *Adv. Opt. Mater.*.

[30] Wen J., Chen L., Chen X. (2021). Use of dielectric metasurfaces to generate deep‐subwavelength nondiffractive bessel‐like beams with arbitrary trajectories and ultralarge deflection. *Laser Photon. Rev.*.

[31] Zhang C., Divitt S., Fan Q. (2020). Low-loss metasurface optics down to the deep ultraviolet region. *Light Sci. Appl.*.

[32] Colburn S., Majumdar A. (2020). Metasurface generation of paired accelerating and rotating optical beams for passive ranging and scene reconstruction. *ACS Photonics*.

[33] Yu B., Wen J., Chen L. (2020). Polarization-independent highly efficient generation of Airy optical beams with dielectric metasurfaces. *Photon. Res.*.

[34] Wen J., Chen L., Yu B. (2021). All-dielectric synthetic-phase metasurfaces generating practical Airy beams. *ACS Nano*.

[35] Fan Q., Zhu W., Liang Y. (2018). Broadband generation of photonic spin-controlled arbitrary accelerating light beams in the visible. *Nano Lett.*.

[36] Li H., Hao W., Yin X., Chen S., Chen L. (2019). Broadband generation of Airy beams with hyperbolic metamaterials. *Adv. Opt. Mater.*.

[37] Yin X., Zhu H., Guo H. (2019). Hyperbolic metamaterial devices for wavefront manipulation. *Laser Photon. Rev.*.

[38] Shi F., Wen J., Lei D. (2020). High-efficiency, large-area lattice light-sheet generation by dielectric metasurfaces. *Nanophotonics*.

[39] Liu W., Zhang Y., Gao J., Yang X. (2018). Generation of three-dimensional optical cusp beams with ultrathin metasurfaces. *Sci. Rep.*.

[40] Porat G., Dolev I., Barlev O., Arie A. (2011). Airy beam laser. *Opt. Lett.*.

[41] Vettenburg T., Dalgarno H. I., Nylk J. (2014). Light-sheet microscopy using an Airy beam. *Nat. Methods*.

[42] Luo W., Xiao S., He Q., Sun S., Zhou L. (2015). Photonic spin Hall effect with nearly 100% efficiency. *Adv. Opt. Mater.*.

[43] Gori F. (1999). Measuring Stokes parameters by means of a polarization grating. *Opt. Lett.*.

[44] Di Domenico G., Ruocco G., Colosi C., DelRe E., Antonacci G. (2018). Cancellation of Bessel beam side lobes for high-contrast light sheet microscopy. *Sci. Rep.*.

[45] Qin F., Huang K., Wu J., Teng J., Qiu C. W., Hong M. (2017). A supercritical lens optical label‐free microscopy: sub‐diffraction resolution and ultra‐long working distance. *Adv. Mater.*.

[46] Qin F., Liu B., Zhu L. (2021). *π*-phase modulated monolayer supercritical lens. *Nat. Commun.*.

[47] He F., Yu J., Tan Y. (2017). Tailoring femtosecond 1.5 μm Bessel beams for manufacturing high-aspect-ratio through-silicon vias. *Sci. Rep.*.

[48] Stoev I. D., Seelbinder B., Erben E. E., Maghelli N. M., Kreysing M. K. (2021). Highly sensitive force measurements in an optically generated, harmonic hydrodynamic trap. *eLight*.

