# Supplementary material for: Broadband generation of accelerating polygon beams with large curvature ratio and small focused spot using all-dielectric metasurfaces
Source: Nanophotonics. 2022 Feb 15;11(6):1203–10. doi: 10.1515/nanoph-2021-0787 (PMC11501288; doi:10.1515/nanoph-2021-0787)
Supplement: Supplementary file 1 — Supplementary Material [file j_nanoph-2021-0787_suppl.docx]

Supporting information for

**Broadband Generation of Accelerating Polygon Beams with Large Curvature Ratio and Small Focused Spot Using All-dielectric Metasurfaces**

Lei Chen^1^, Saima Kanwal^1^, Yongzheng Lu^1^, Dawei Zhang^1^, Xu Chen^1^, Jian Chen^2^ and Jing Wen^1^*

^1^Engineering Research Center of Optical Instrument and Systems, Ministry of Education and Shanghai Key Lab of Modern Optical System, University of Shanghai for Science and Technology, No. 516 Jun Gong Road, Shanghai. 200093, China

^2^ School of Optical-Electrical and Computer Engineering, University of Shanghai for Science and Technology, Shanghai 200093, China

*Corresponding Author: jwen@usst.edu.cn

**Simulation results of the accelerating polygon beams**

**Ⅰ. Simulation results of the accelerating polygon beams**

Figure S1 shows the simulated intensity distributions of quadrilateral beams with *m* = 4 and hexagonal beams with *m* = 6 at *λ* = 500, 532, 580, 633, 710, 780, and 850 nm at various propagation distances. The phase for generating quadrilateral beams with *m* = 4 and hexagonal beams with *m* = 6 are shown in Figures 2c and 2d, respectively. The four or six off-axis identical lobes of PBs gradually move away from the optical axis with increasing wavelength, which agrees well with the experimental results.

Figure S2 shows the simulated longitudinal field distributions of the quadrilateral beam at *λ* = 500, 532, 580, 633, 710, 780, and 850 nm. The simulation results show that the curvature ratio of the PBs increases gradually with the increase of the wavelength, which is consistent with the experimental results.

Figure S3 shows the simulated longitudinal needle-like field distributions and the extracted transverse fields at the focal plane at *λ* = 500, 532, 580, 633, 710, 780, and 850 nm, respectively. As shown in Figure S3(a1–a7), the depths of focus of the needle-like region are 92, 87, 80, 73, 65, 59 and 54 μm at *λ* = 500, 532, 580, 633, 710, 780 and 850 nm, respectively, which are close to the experimental results 87, 83, 77, 72, 60, 56 and 53 μm. As shown in Figure S3(b1–b7) and (c1–c7), the simulated FWHMs at *λ* = 500, 532, 580, 633, 710, 780 and 850 nm are 2.66, 2.66, 2.65, 2.64, 2.62, 2.61 and 2.24 μm, respectively.


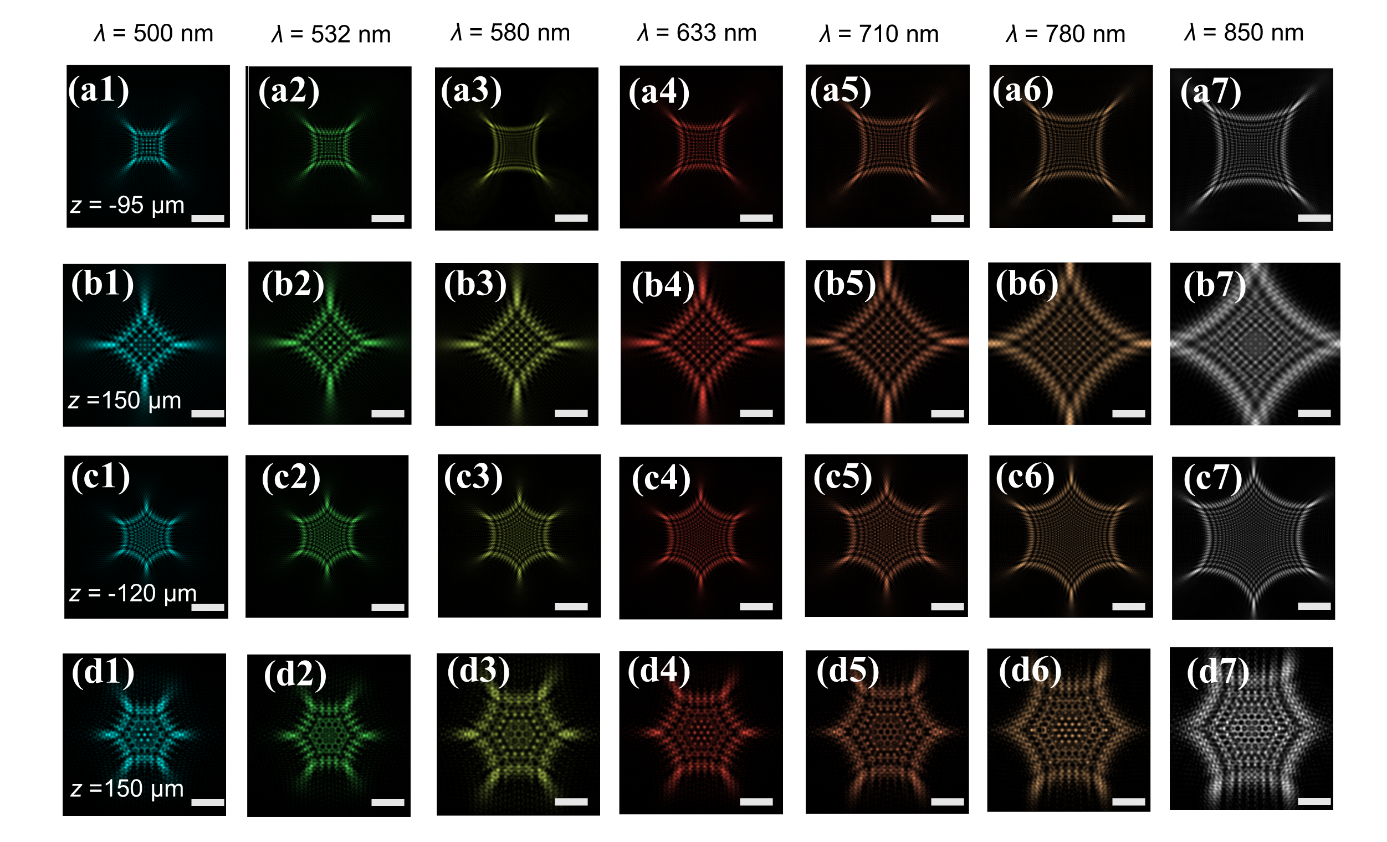


**Figure S1.** Simulated intensity distributions of the PBs in *x*-*y* plane. Simulated intensity distributions of the generated quadrilateral beams (a1–a7) for *z* = -95 μm and (b1–b7) for *z* = 150 μm at *λ* = 500, 532, 580, 633, 710, 780 and 850 nm, respectively while, simulated intensity profiles of the generated hexagon beams (c1–c7) for *z* = -120 μm and (d1–d7) for *z* = 150 μm at *λ* = 500, 532, 580, 633, 710, 780 and 850 nm, respectively. Scale bar = 17 μm (a1­–a7, b1–b7) and 20 μm (c1–c7, d1–d7). All figures are false-colored images.


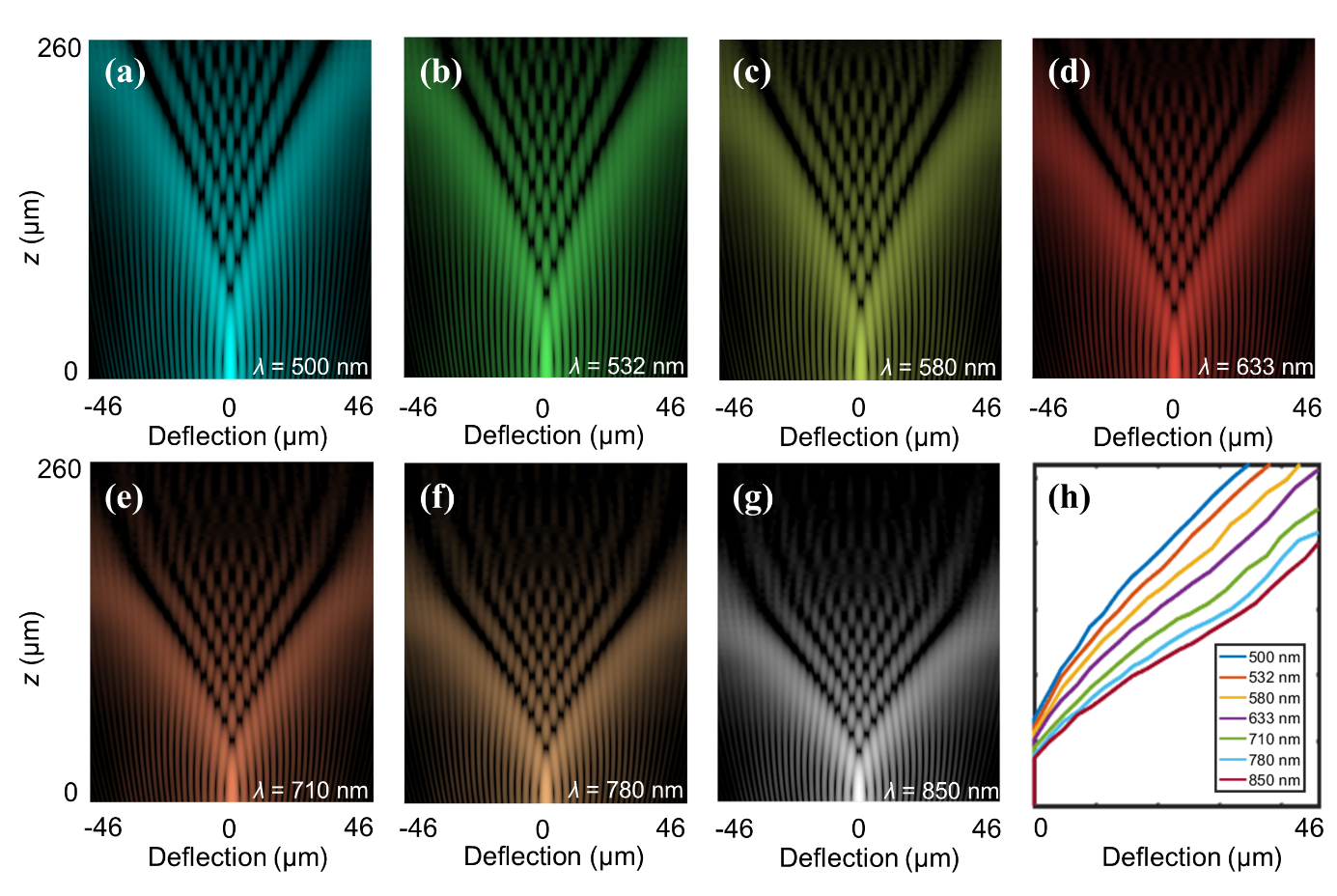


**Figure S2.** Simulated longitudinal intensity distributions of the PB. (a–g) Simulated longitudinal optical intensity profiles for the PB with *m* = 4 at *λ* = 500, 532, 580, 633, 710, 780, and 850 nm, respectively. To facilitate visualization, the patterns in (a–g) are false-colored images in the log scale. (h) Simulated beam deflection of the main accelerating channel along the longitudinal direction for the above wavelengths.


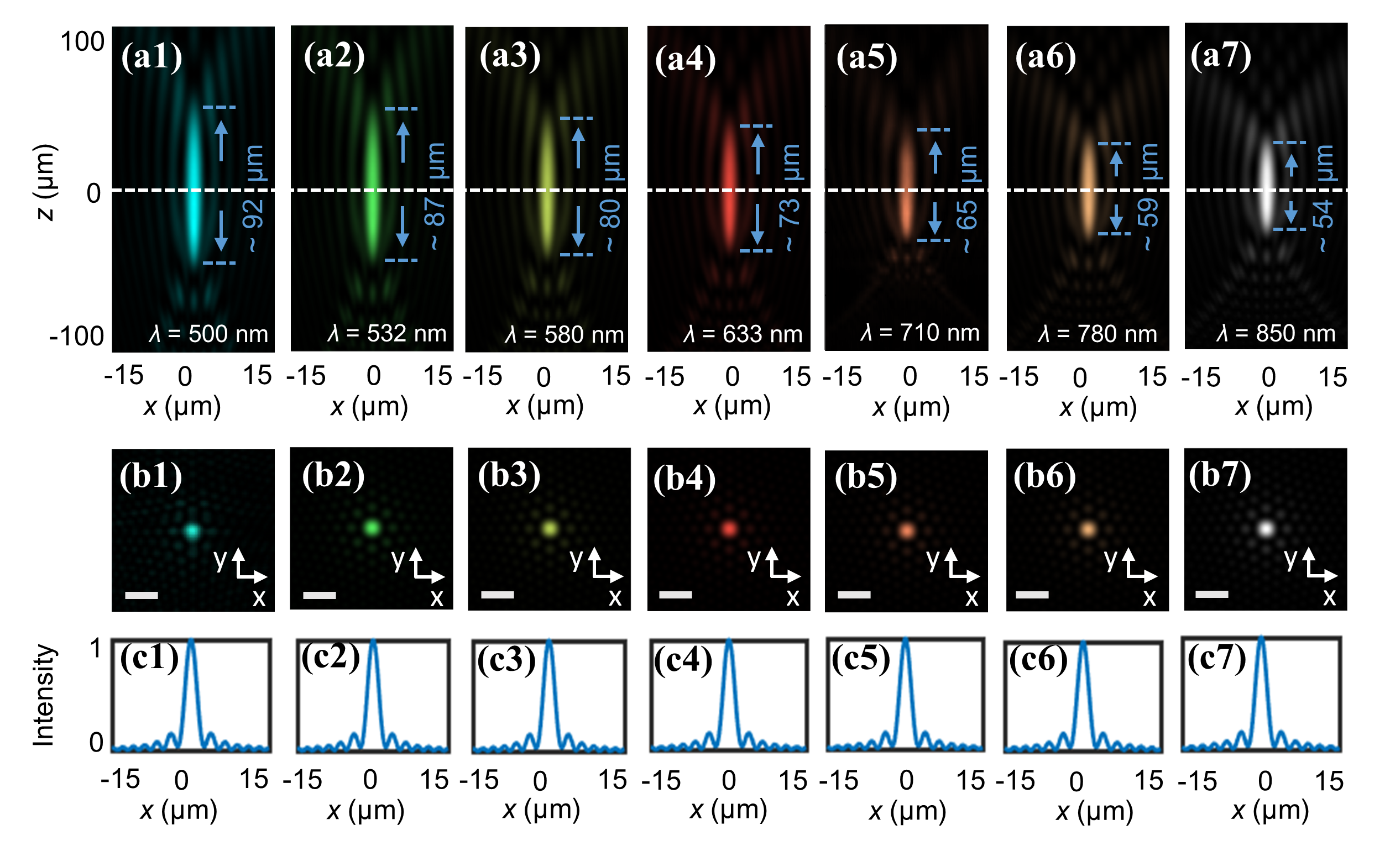


**Figure S3.** Simulated intensity distributions of the PB in the needle-like region. (a1–a7) Simulated intensity distributions of the PB (*m* = 4) at the *x*-*z* plane and (b1–b7) simulated transverse field distributions at the focal plane (the *x*-*y* plane) of the PB at *λ* = 500, 532, 580, 633, 710, 780 and 850 nm. To facilitate visualization, the patterns in (a1–a7) and (b1–b7) are false-colored images. (c1–c7) Normalized intensity profiles along the horizontal axes at the center of the focal spots extracted from (b1–b7). Scale bar = 6 μm.

**Ⅱ. Control the depth of focus of the needle-like region of the accelerating polygon beams**

Figure S4 shows the phase profiles to generate the PBs (*m* = 4) with different depths of focus at *λ* = 650 nm. To demonstrate the method to control the depth of focus of the needle-like region, we keep the focal length *f* of the Fresnel lens at 50 μm at *λ* = 650 nm and vary the values of *C* to 25000, 30000, and 35000, respectively. The phases used to generate the PBs in K space are shown in Figures S4(a–c). All phase diagrams in Figure S4 are 50 μm in diameter.

Figures S5(a–c) show the simulated longitudinal intensity distributions of the PBs (*m* = 4) at *λ* = 650 nm. The corresponding phases imposed on the metasurface are shown in Figures S4(d–f). Once the focal length *f* of the Fresnel lens is determined, the depths of focus of the needle-like regions can be adjusted by changing the value of *C*. The depths of focus of the needle-like regions shown in Figures S5(a–c) are 19.6, 18.3, and 16.7 μm, respectively, which decrease with decreasing *C* value. It is worth noting that zʹ is defined as the distance of the *x*-*y* plane from the metasurface.

Figure S6a exhibits the magnitude and the polarization direction of the simulated electric fields of the PB (*m* = 4) at *λ* = 650 nm. The corresponding phase imposed on the metasurface is shown in Figure S4d. Figure S6b shows the magnitude and direction of the simulated energy flow of the PB. As depicted in Figure S6b, most of the energy flow in the needle region points towards the *z*-direction.


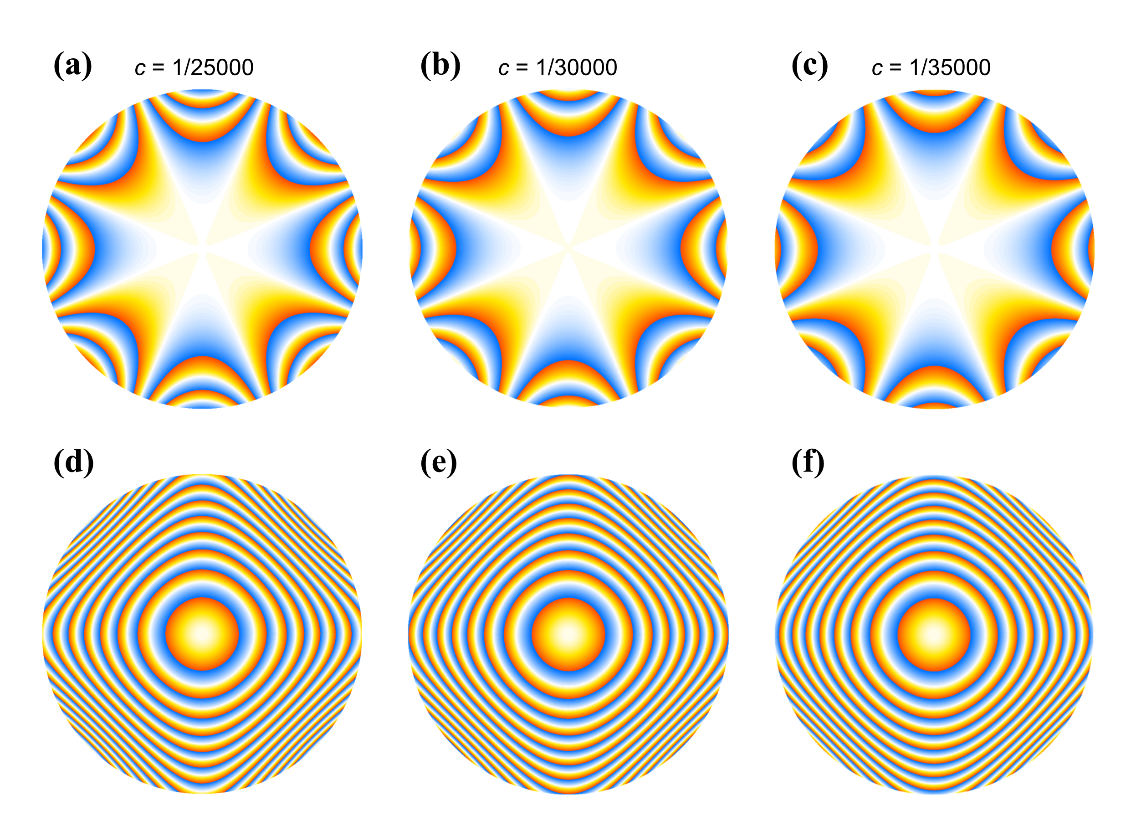


**Figure S4.** Phase design for generating PBs. Phase profiles to generate quadrilateral beams with *C* = 1/25000 in K space (a) and imposed on the metasurface (d), with *C* = 1/30000 in K space (b) and imposed on the metasurface (e), with *C* = 1/35000 in K space (c) and imposed on the metasurface (f).


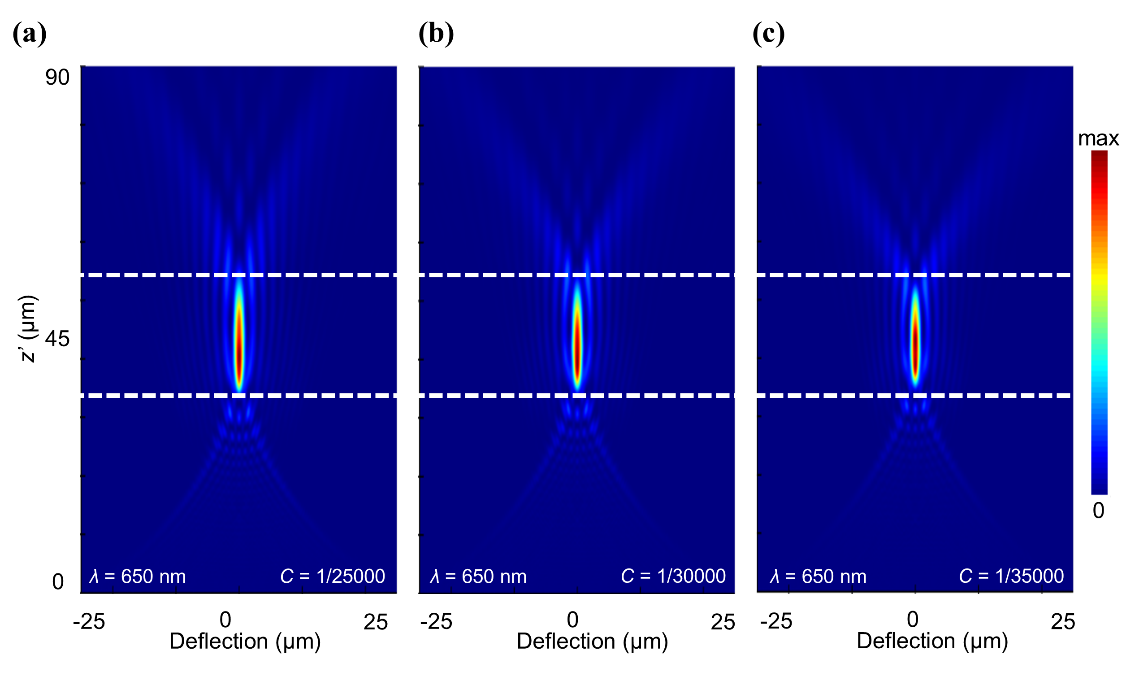


**Figure S5.** Simulated longitudinal intensity distributions for different parameters *c*. Simulated longitudinal intensity distributions of the PBs (*m* = 4) at *λ* = 650 nm with *C* = 1/25000 (a), 1/30000 (b), and 1/35000 (c).


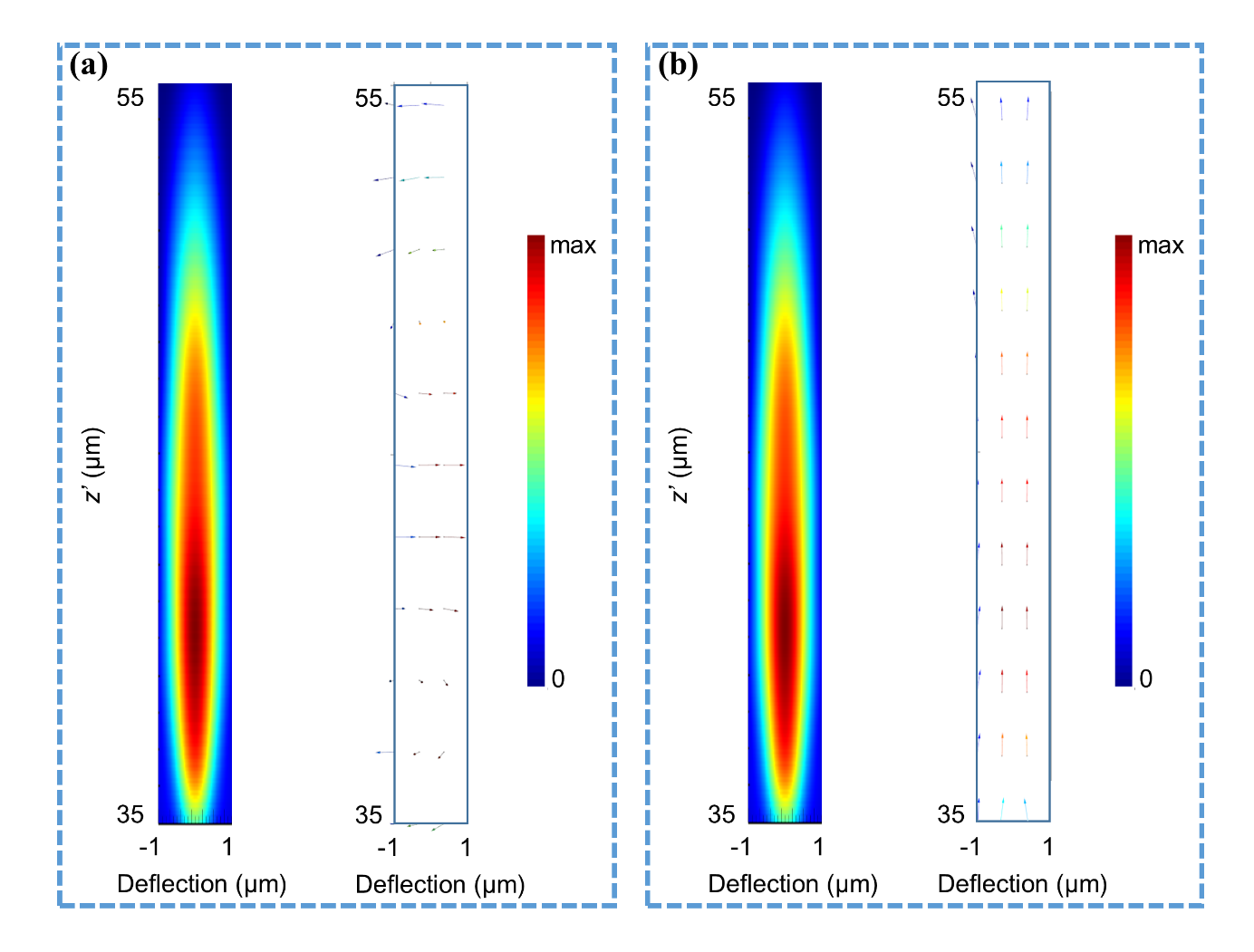


**Figure S6.** Simulated polarization and energy flow states in the needle-like region. The simulated magnitude and the polarization direction of the electric field distribution (a) of the PB (*m* = 4) as well as the magnitude and direction of the Poynting vector (b) of the PB (*m* = 4) at *λ* = 650 nm.

**Ⅲ. Simulated results of the accelerating polygon beams with *m* = 1**

Figure S7b shows the phase of a Fresnel holographic lens with a focal length *f* = 50 μm at *λ* = 650 nm. Figure S7c shows the phase profile to generate the PB with *m* = 1 in K space (*C* = 1/5). Figure 7a presents the phase mask imposed on the metasurface (addition of Figure S7b and Figure S7c) to generate the PB with *m* = 1. Figure S7d shows the simulated longitudinal intensity distribution of the PB at *λ* = 650 nm. The green dashed lines in Figure S7d represent the contour of the outermost periphery of the propagation of PBs in the longitudinal plane. It can be seen from Figure S7d that the PB spreads to both sides along the inclined straight lines during propagation. Figures S7(e–h) show the electric field distributions of the PB in the *x*-*y* plane at *z* = 0, 5,10, and 15 μm, respectively.


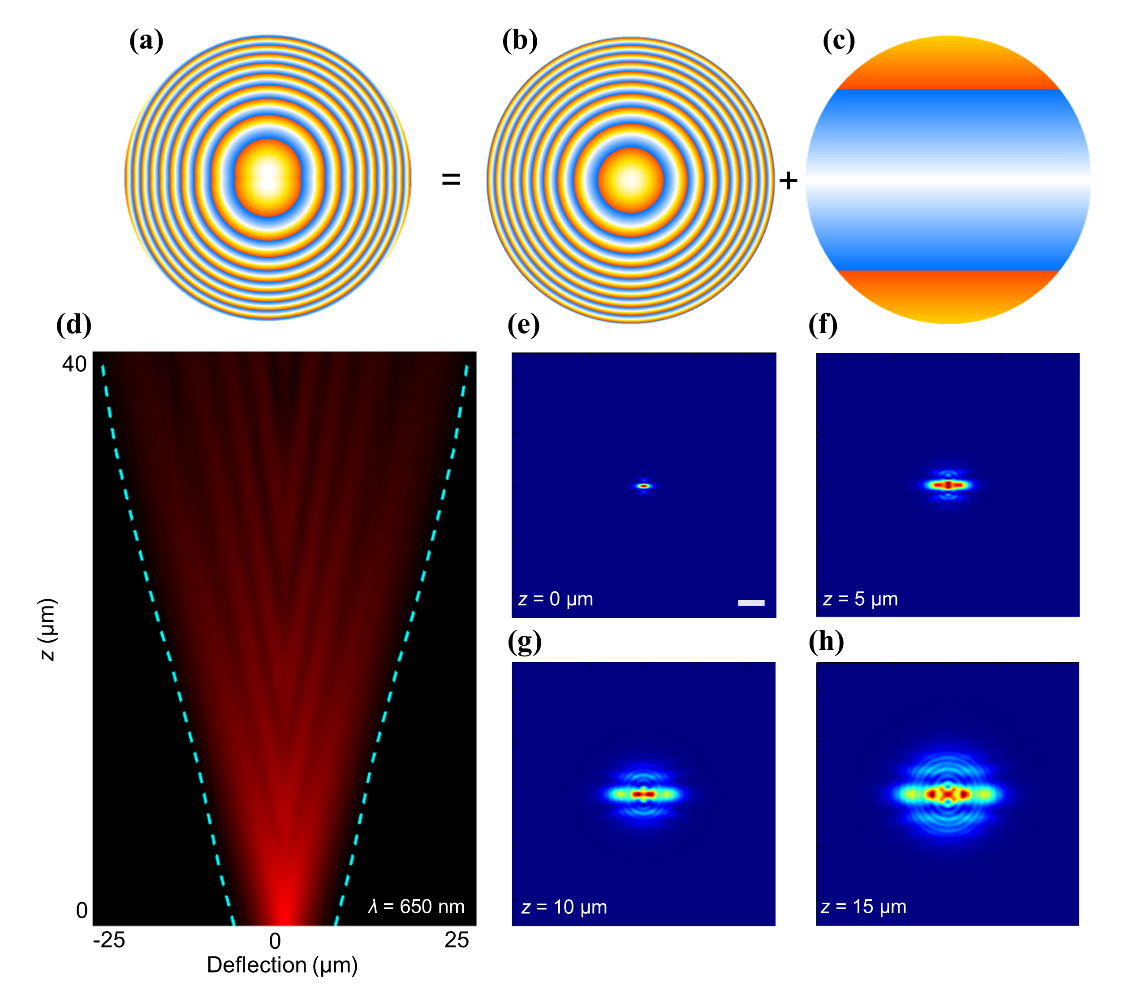


**Figure S7.** Simulated results of the PB with m = 1. (a–c) Phase profile (a) to generate the PB with *m* = 1 is the addition of (b) and (c). (d–h) Simulated electric field distributions in the longitudinal plane (d) and the transverse plane at different *z* positions (e–h) of the corresponding PB at *λ* = 650 nm. To facilitate visualization, the pattern in (d) is a false-colored image in the log scale.
